# Computation of Förster Resonance Energy Transfer in Lipid Bilayer Membranes

**DOI:** 10.1021/acs.jpca.2c04524

**Published:** 2022-10-19

**Authors:** Richard Jacobi, David Hernández-Castillo, Novitasari Sinambela, Julian Bösking, Andrea Pannwitz, Leticia González

**Affiliations:** † Institute of Theoretical Chemistry, Faculty of Chemistry, University of Vienna, Währinger Straße 17, 1090Vienna, Austria; ‡ Doctoral School in Chemistry (DoSChem), University of Vienna, Währinger Straße 42, 1090Vienna, Austria; ¶ Institute of Inorganic Chemistry I, Ulm University, Albert-Einstein-Allee 11, 89081Ulm, Germany; § Vienna Research Platform on Accelerating Photoreaction Discovery, University of Vienna, Währinger Straße 17, 1090Vienna, Austria

## Abstract

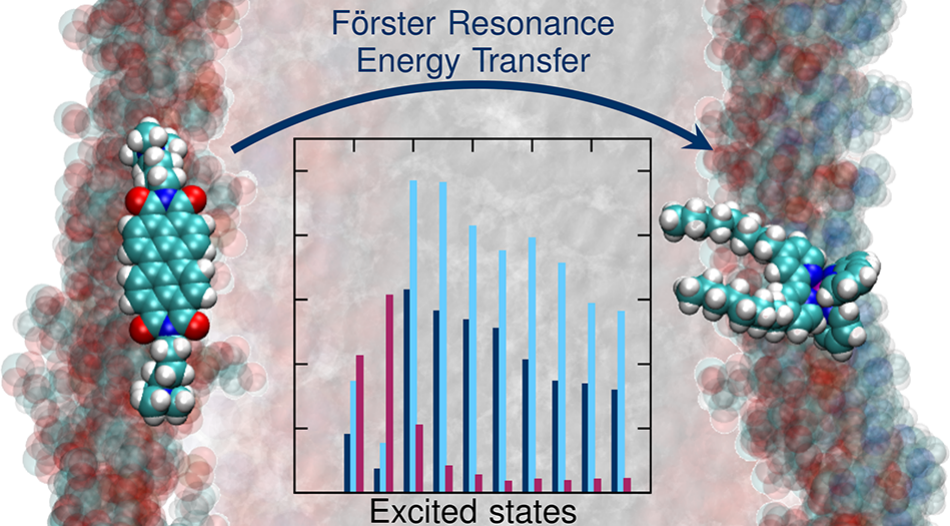

Calculations of Förster Resonance Energy Transfer (FRET) often neglect the influence of different chromophore orientations or changes in the spectral overlap. In this work, we present two computational approaches to estimate the energy transfer rate between chromophores embedded in lipid bilayer membranes. In the first approach, we assess the transition dipole moments and the spectral overlap by means of quantum chemical calculations in implicit solvation, and we investigate the alignment and distance between the chromophores in classical molecular dynamics simulations. In the second, all properties are evaluated integrally with hybrid quantum mechanical/molecular mechanics (QM/MM) calculations. Both approaches come with advantages and drawbacks, and despite the fact that they do not agree quantitatively, they provide complementary insights on the different factors that influence the FRET rate. We hope that these models can be used as a basis to optimize energy transfers in nonisotropic media.

## Introduction

Oxygen-producing photosynthesis is a process resulting from the interplay of many subunit protein complexes that are embedded into the thylakoid membranes of chloroplasts.^1,2^ Energy and charge transfer between the individual units are essential for the capture and subsequent funneling of the excitation energy to the catalytic reaction centers. Therefore, to understand and ultimately mimic natural photosynthesis, it is important to develop computational protocols which are able to investigate these processes.

A useful tool for the investigation of energy transfer processes between chromophores is Förster Resonance Energy Transfer (FRET) theory.^3−5^ Here, energy is transferred from an electronically excited-state donor to an acceptor chromophore in the electronic ground state, thus producing a ground-state donor and an excited-state acceptor. This transfer occurs as a result of radiationless resonance between the transition electric dipole moments (TDMs) of the respective states in donor and acceptor. Since, within this methodology, the coupling is represented as a dipole–dipole interaction, FRET theory holds between donor and acceptor molecules sufficiently separated such that the electronic wave functions do not overlap and at interchromophoric distances smaller than the excitation wavelength, such that emission and reabsorption do not occur.^6^

However, there are different mechanisms which lead to a decay of the excited state of the donor chromophore and therefore compete with FRET. These include prominently radiative decay, in the case of organic molecules usually fluorescence, and nonradiative relaxation pathways to the ground state. Furthermore, charge transfer processes can play an important role as they can mediate photodegradation of the chromophores, or transfer energy through an alternative channel, e.g., Dexter energy/electron transfer.^7^ Since these processes only occur at shorter distances, i.e., below at least 15 Å, they can be mostly neglected in FRET studies.

Within the FRET formalism, the energy transfer rate depends on the interchromophoric distance, the magnitude and orientation of the TDMs, as well as on the spectral overlap. In particular, the high sensitivity to distance renders FRET a powerful tool to measure the spatial separation of chromophores, i.e., as a molecular ruler.^8,9^ However, FRET theory as a distance measuring device should be applied with care, as orientational anisotropy and the breakdown of the point–dipole approximation, especially at small distances, can have significant impact on the energy transfer efficiency.^10^ In spite of this, both experimental and theoretical studies tend not to include all factors in their analysis. The dependency of κ^2^—the orientation factor of the TDMs on different supramolecular arrangements—is often neglected in experimental approaches due to the not straightforward assessment of the orientation of the chromophores. κ^2^ is then usually averaged assuming isotropic arrangements.^11^ This approximation, however, does not hold true in media which constrain the reorientation of subunits, such as protein complexes or membrane environments,^9^ resulting in errors in the calculated distances of well over 10 Å.^11,12^ Theoretical investigations are able to closely monitor the orientational dependencies. However, they commonly neglect the influence of the spectral overlap, either by computing only the intermolecular interaction factor directly,^13,14^ estimating the FRET efficiency against the fixed Förster radius,^15^ or by using experimentally recorded data for the overlap.^6^

**Figure 1:**
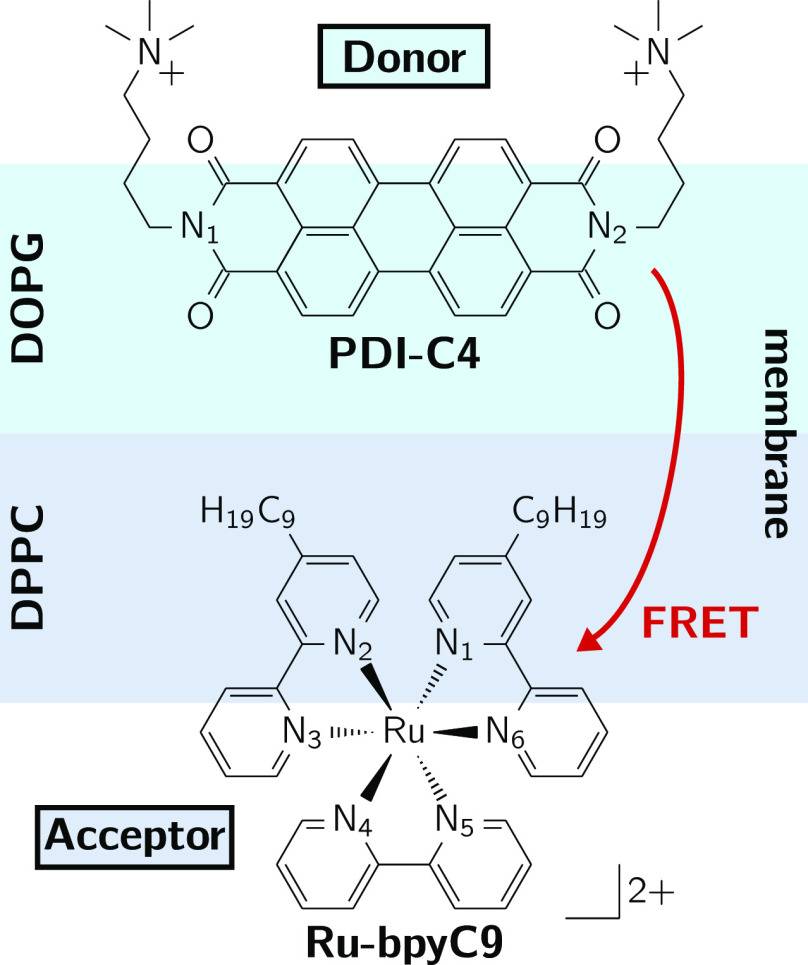
Artificial bioinspired light-harvesting system. The PDI-C4 energy donor and Ru-bpyC9 acceptor molecule are embedded into a lipid bilayer membrane consisting of DOPG and DPPC lipid molecules in the upper and lower leaflets, respectively.

In this work, we present two computational protocols to estimate the FRET rate and test their performance on an artificial bioinspired light-harvesting system, in order to provide a computational framework with which energy transfer of complex chromophoric systems in anisotrpoic media can be studied and analyzed. In particular, we embed an N-substituted perylene diimide (PDI-C4) as energy donor alongside a modified Ru(II)–tris(bipyridine) (Ru-bpyC9) metal complex that acts as the energy acceptor, in a lipid bilayer membrane comprising dioleoylphosphatidylglycerol (DOPG) and dipalmitoylphosphatidylcholine (DPPC) lipids (see Figure 1). The use of lipid bilayers, for instance, the spherical membranes of liposomes, is a promising approach toward mimicking natural photosynthesis, as they allow one to confine redox half-reactions, facilitate charge separation, and avoid cross-reactivity.^16,17^ This approach has been previously employed by some of us.^18^ The selected donor molecule is derived from a well-studied organic chromophore, perylene diimide, while the acceptor is the functionalized ruthenium(II) tris(bipyridine) model photosensitizer, one of the most widely employed photosensitizers.

We base our approaches on the topology and the photophysical properties of the systems, i.e., the distance between the chromophores, magnitude, and orientation of the TDMs, as well as the spectral overlap, in order to compute the FRET rate constant of the energy transfer between the chromophores. In the first approach, we combine quantum mechanical (QM) calculations to obtain the photophysical properties of the chromophores in implicit solvation with classical molecular mechanics (MM) molecular dynamics (MD) simulations to assess the position and alignment of the chromophores within the lipid bilayer membrane. In the second approach, we directly compute the emission and absorption spectra of the chromophores as well as the TDMs directly within the membrane environment by means of hybrid QM/MM calculations. Our results illustrate that both approaches exhibit strengths and weaknesses, but they complement each other to derive a concise theoretical picture that serves for the analysis and optimization of the energy transfer between the chromophores within the membrane.

## Theory

### Förster Resonance Energy Transfer

Energy transfer between two chromophores sufficiently spatially separated, such that their wave function overlap can be neglected, was described by Theodor Förster in 1946.^3,4^ In this context, the intermolecular interaction *V*_DA_ between the excited state donor and ground state acceptor are of electrostatic nature and can be modeled as field interactions of two dipoles:^6^
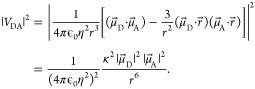
1Here,  and  are the TDMs of the respective donor and acceptor,  is the distance vector with its magnitude *r*, while ϵ_0_ and η are the vacuum permittivity and medium refractive index, respectively. In this work, we used the experimentally measured refractive index in DPPC monolayers of η = 1.478.^19^ The alignment dependence of the dot products between TDMs and the distance vector can be collected in the orientation factor κ^2^, which ranges from 0 to 4 and is usually approximated as ^2^/_3_ in isotropic media.^11^

The energy transfer rate *k*_FRET_ within this formalism is then given as
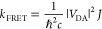
2where *J* is the spectral overlap of donor fluorescence and acceptor absorption spectra, both normalized to the unit area:
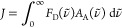
3Accordingly, the computation of the FRET rate requires knowledge of the TDMs  and  and the distance vector , as well as the donor emission and acceptor absorption spectra,  and , respectively. These quantities can be estimated in different ways. Here, we consider two approaches. In the first, which we call the *two-step* approach, we compute the spectra and TDMs of the chromophores in implicit solution and then use linear combinations of atomic position vectors as a representation for the TDM orientation to track their alignment during classical MM-MD simulations of the chromophores in the membrane. These classical MD simulations also serve to evaluate the distance between the donor and acceptor. In the second, coined as the *one-step* approach, we directly compute spectra and TDMs within the membrane environment using an electrostatic embedding QM/MM methodology.

## Computational Details

### Quantum Mechanical Calculations

Long aliphatic chains can adopt numerous different conformations, rendering it insufficient to merely compute the properties of one conformer.^20−22^ Therefore, all computations of properties in implicit solvent are performed on a conformational ensemble of 100 or 92 structures for PDI-C4 and Ru-bpyC9, respectively. Details on the ensemble generation can be found in the Supporting Information, Section S1.1.

The photophysical properties of the donor and acceptor chromophores are calculated using density functional theory (DFT) and its time-dependent version (TD-DFT) at the RB3LYP/def2-SVP level of theory, as implemented in the program package Gaussian 16, revision C.01.^23^ Ground state geometries are obtained with DFT and electronic excited states with TD-DFT. In the latter cases, 30 singlet excited states are calculated. Solvent effects for acetonitrile are included implicitly where noted using the polarizable conductor-like calculation model^24,25^ by placing the solute in a cavity within the solvent reaction field. Within the framework of the vertical approximation, properties related to absorption of radiation, i.e., the absorption spectra and the corresponding TDMs, are computed on the optimized electronic ground state structure, while emissive properties are computed on the optimized first singlet excited S_1_ state geometry, assuming that higher electronic excited states eventually relax into the S_1_ state prior emission.^26^ Dispersion interaction effects are corrected empirically using Grimme’s D3 model with Becke–Johnson damping.^27,28^ All geometry optimizations are performed with tighter cutoffs on forces and step size (tight keyword in Gaussian 16). The convergence of the geometry optimizations is confirmed by the absence of imaginary frequencies within the harmonic approximation.

Wave function analysis is performed with Multiwfn, version 3.7,^29^ using the results of the Gaussian 16 calculations to compute the TDMs. Where noted, vibrationally resolved spectra are computed using the Franck–Condon Herzberg–Teller (FCHT) method developed by Barone and co-workers^30,31^ as implemented in Gaussian 16. This method computes the nuclear wave functions within the harmonic approximation as well as the change of TDMs *w.r.t.* the normal coordinates of the molecule in a first order approximation to account for symmetry-allowed and symmetry-forbidden transitions. Accordingly, normal modes are computed both on the ground state and the first excited state structures. Standard TD-DFT and FCHT spectra are convoluted from the vertical transitions using Gaussian functions. We use a full width at half-maximum (fwhm) of 0.08 eV for PDI-C4 and 0.6667 eV for Ru-bpyC9 in the implicit solvation case to best match the experimental spectra. Note that the fwhm of PDI-C4 is small as nuclear motion is included in the FCHT method, while in the case of Ru-bypC9 larger arbitrary fwhm is needed to resemble the experimental line shape.

### Molecular Dynamics Simulations

To elucidate the orientation and position of the chromophores in the lipid bilayer and generate starting structures for subsequent hybrid QM/MM calculations, MD simulations are performed on the chromophores embedded into a lipid bilayer membrane.

For each chromophore, different insertion modes and depths are investigated in individual systems. Which and why the specific positions were chosen, is discussed in the results section. The chromophore and its initial arrangement have an impact on the size of the periodic box and the number of lipids. Details on the system generation are presented in the Supporting Information, Section S1.2, and the nuclear coordinates of the chromophores’ initial geometries are included in a zip file as additional Supporting Information. In general, the box dimensions of the initial structures are around 80 Å in the *x*- and *y*-directions, which span the plane in which the membrane is assembled, and are around 95 Å in the *z*-direction. The leaflet, in which PDI-C4 is embedded, consists of DOPG lipids, while the Ru-bpyC9 leaflet consists of DPPC molecules. The entire systems of the chromophores in the membrane are included as Supporting Information.

Prior to production, the systems are minimized, heated, and equilibrated. The simulation protocol is described in detail in the Supporting Information, Section S1.2.

Each trajectory is analyzed with respect to the distance between chromophores’ center of mass and the center of the membrane as represented by the center of mass of the final atom in the DOPG tail groups. In all systems, the membrane is assembled and remained in the *xy*-plane, so that the angle of the TDM to the surface of the membrane is evaluated as the angle *w.r.t.* the *xy*-plane.

### Hybrid QM/MM Calculations

For the QM/MM simulations, we limit the analysis to two starting insertion modes for each chromophore and select an MM-MD trajectory of 100 ns per insertion mode. For the emitter PDI-C4, we perform the trajectory production with excited state RESP charges to equilibrate the solvent to the excited state electrostatics. We select 50 snapshots per trajectory, so that in the end we attain 100 structures for each chromophore. Each of these starting conformations are propagated for a random time between 150 and 200 ps on the QM/MM level to avoid all the geometrical parameters to coherently converge to the same values while relaxing from the force field to the QM level of theory. The chromophores are described at the RB3LYP/def2-SVP level of theory, as implemented in Terachem, version 1.9.2018.07-dev,^32−34^ while the environment is represented using MM. Ru-bpyC9 is propagated in the ground state and PDI-C4 in the first electronic excited state.^35^ Effective core potentials are used for Ruthenium.^36^ Spectra and TDMs are computed on the final structures including the MM environment represented as point charges using the Gaussian16 workflow described above. For the convolution of the QM/MM spectra, we use Gaussian functions with fwhm of 0.05 eV for PDI-C4 and 0.2 eV for Ru-bpyC9.

### Experimental Details

In this work, computed spectra are compared to experimentally recorded spectra. Information about the synthesis of the chromophores and the spectroscopic measurements can be found in the Supporting Information, Section S2.

## Results and Discussion

**Figure 2:**
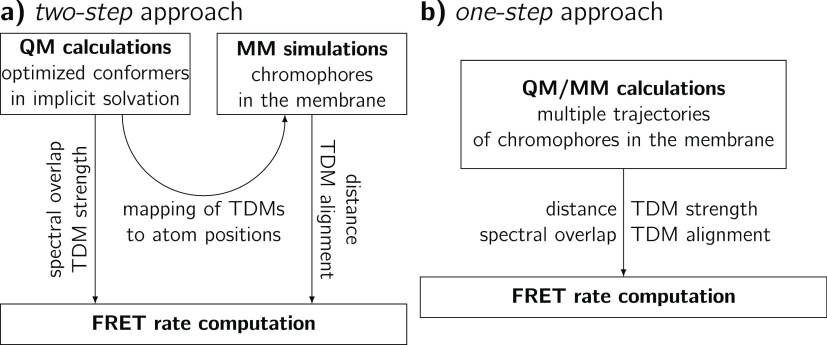
Workflows of the (a) *two-step* and (b) *one-step* FRET rate calculation approaches.

The energy transfer rate between the chromophores within the realm of FRET theory is calculated in two ways. In the *two-step* approach (Figure 2a), we compute the absorption and emission spectra as well as the TDMs of the two chromophores using TD-DFT in implicit solvation and then map the actual TDM orientation in terms of linear combinations of atomic positions. This mapping enables us to superimpose the TDMs obtained from QM calculations onto purely classical MD simulations, which yields the position and orientation of the chromophores, and thus the respective TDMs within the lipid bilayer membranes. The FRET rate computation results then from the combination of the quantum chemical and classical calculations. In the other, *one-step* approach (Figure 2b), we use QM/MM hybrid calculations to directly compute the photophysical properties within the explicit environment of the membrane. As it will be shown, none of the two approaches is perfect; however, the comparison of both approaches allows for a more robust evaluation of the energy transfer efficiency, as drawbacks of one method are covered by the other and *vice versa*.

### *Two-Step* FRET Calculation

The computation of the FRET rate in this *two-step* approach is based on the assumption that the photophysical properties, i.e., absorption and emission spectra, as well as the TDMs are sufficiently similar in implicit solution and in the membrane environment. Within this approximation, in the first step, we compute the spectral overlap for the isolated molecules using TD-DFT, and in a second step perform an MM-MD simulation to get information about the distance between the chromophores and their relative orientation. The one quantity bridging the classical MD simulations and the quantum chemical TD-DFT is the TDM, as this is a property derived from the electron distribution and hence inaccessible from purely classical simulations. Nevertheless, its alignment can only be obtained within the membrane and thus from the MD simulations. Accordingly, we compute the TDMs from TD-DFT and map their orientation onto the molecular geometry by expressing them as linear combination of atomic position vectors.

**Figure 3:**
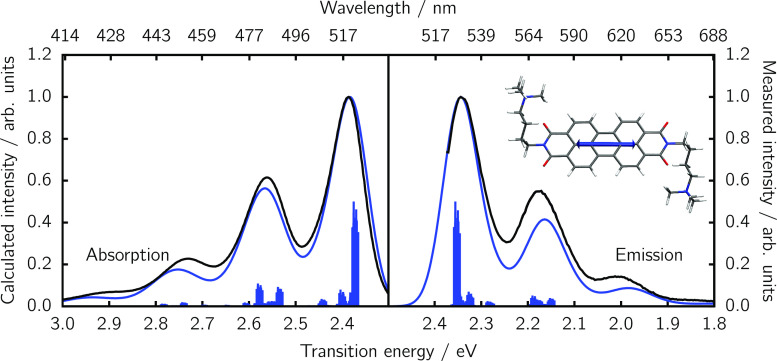
Computed absorption and emission spectra of the donor PDI-C4 (blue lines) computed from an ensemble of 100 geometries versus the experimental counterpart (black lines). For a better comparison to the experiment, computed spectra were blue-shifted by +0.44 eV and +0.42 eV for absorption and emission, respectively. Individual S_1_ vertical excitation energies are shown with impulses. The S_1_ → S_0_ TDM for the most stable conformer is shown as an inset.

Vibrationally resolved spectra are calculated using the FCHT method^30,31^ on the initial ensemble of 100 optimized PDI-C4 conformers. Figure 3 shows the absorption and emission spectra resulting from a Boltzmann weighted sum of the spectra of each individual conformer. They are obtained according to the Boltzmann distribution, multiplying the property value *p*_*i*_ with its weight *w*_*i*_ and summing over the entire ensemble of *N* structures, which results in the expectation value for the ensemble ⟨*p*⟩:
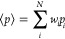
4For the spectra, the quantity to weight is the intensity of the individual transitions. Subsequently, a single spectrum is convoluted from the weighted intensities.

Since the absorption signal of PDI-C4 below 3 eV is dominated by the S_0_ → S_1_ transition, it is sufficient to include the overlap between the nuclear modes of these two electronically excited states in the FCHT calculation. The same is true for the emission spectrum, as according to Kasha’s rule^26^ fluorescence occurs from the energetically lowest singlet state; i.e., it corresponds to the S_1_ → S_0_ transition. The nature of the *S*_1_ excited state of the donor is discussed in the Supporting Information, Section S3.1.

As often done in the literature when spectra are computed from TD-DFT,^37−39^ we need to shift our FCHT spectra by 0.44 and 0.42 eV (for absorption and emission, respectively) in order to match the experimental maximum bands. Previous benchmarks have shown that, for similar perylene diimide-based molecules, 0–0 energies can vary between different functionals by more than 0.6 eV.^40^ After the shifting, one can see that the agreement between the experimental and computed profiles (Figure 3) is excellent, validating the chosen level of theory employed to describe the donor PDI-C4.

In most of the conformers of the ensemble, the TDM for the S_1_ → S_0_ transition of the donor, , aligns almost perfectly with the N_1_–N_2_ (recall Figure 1) connecting vector, which is denoted . Within the ensemble, the largest deviation of the angle between  and  is slightly over 1°. Therefore, we deem  as appropriate to approximate the orientation of  during the MD simulations and, in consequence, map  onto . For the FRET rate computation,  is scaled to equal the dipole strength of  Boltzmann weighted for the conformational ensemble at 23.8 au^2^ (12.3 D). Since energy transfer does not only occur from the S_1_ minimum geometry but could also occur from the vicinity of the Franck–Condon region, i.e., the excited state potential at ground state geometry, we compare the TDMs at both S_0_ and S_1_ conformational ensembles. Both sets have TDMs almost identical in orientation, although the TDMs at the Franck–Condon region are of reduced magnitude (with values ranging from 15.6 to 16.3 au^2^) compared to those at the S_1_ minimum. Thus, for the computation of the FRET rates, we only include the TDMs computed at the S_1_ minimum geometries.

**Figure 4:**
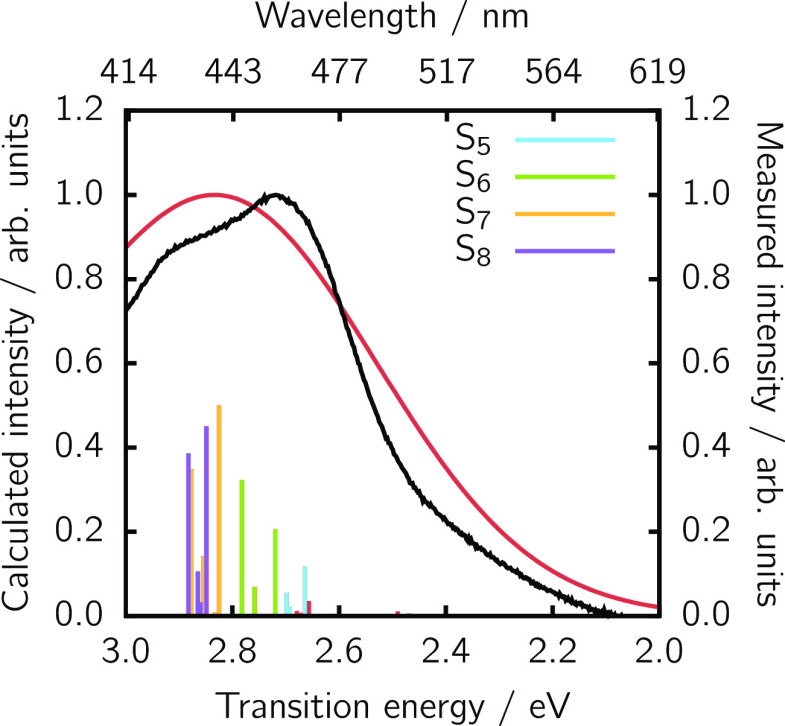
Computed absorption spectrum of the acceptor Rubpy-C9 (red line) based on an ensemble of 92 structures versus the experimental counterpart (black line). Vertical excitation energies are shown with impulses. Individual S_5_ to S_8_ excitations are color coded.

For the acceptor Ru-bpyC9, the conformational ensemble consists of 92 structures from which the Boltzmann weighted TD-DFT spectrum is shown in Figure 4. In this case, the energetic agreement with the experimentally recorded spectrum as well as with previous computations on the Ru(II)–tris(bipyridine) core unit^41^ is very good, requiring no shifting. Interesting, the bright part of the spectrum (below 3 eV) is dominated by four distinct excited states, the S_5_, S_6_, S_7_, and S_8_, which will be used for the calculation of the FRET rate. The nature of the four relevant excited states of the acceptor is discussed in the Supporting Information, Section S3.2.

**Figure 5:**
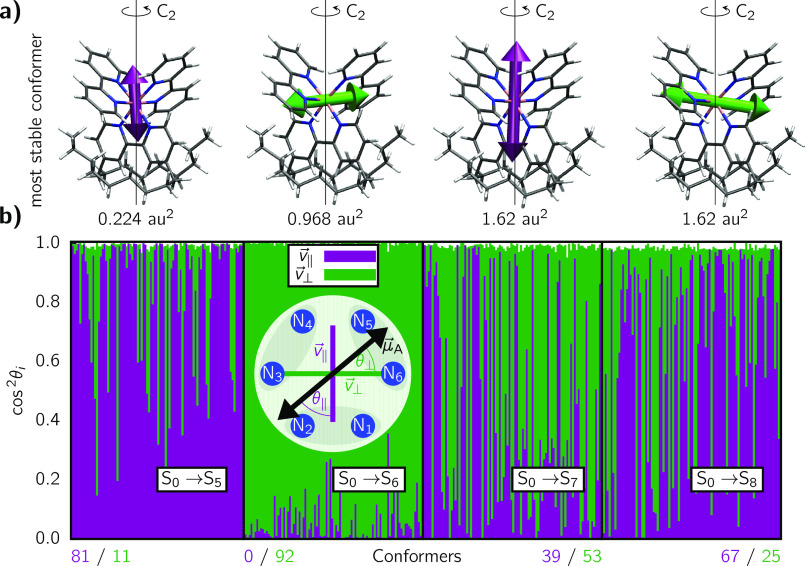
(a) Orientation of the transition dipole moments corresponding to the S_0_ → S_5_, S_0_ → S_6_, S_0_ → S_7_, and S_0_ → S_8_ transitions for the most stable conformer of the Ru-bpyC9 acceptor chromophore. The expectation values of the TDM strength Boltzmann weighted for the ensemble is given underneath the conformers. (b) Plots of cos^2^ θ_*i*_ for each of the transitions in the conformational ensemble. The numbers below the plot indicate the number of conformers for which either  (violet) or  (green) character dominates. Conformers are ordered from left to right, starting with the most stable conformer. The inset indicates the definition of θ_∥_ and θ_⊥_.

The TDMs of these four transitions are shown in Figure 5a for the most stable conformer of the ensemble. As it can be seen, the TDMs orient either almost parallel to the *C*_*2*_ symmetry axis of the complex ( and , violet in Figure 5a), or approximately perpendicular ( and , in green). However, it is necessary to monitor the alignment of the TDMs for all the conformers of the ensemble, in order to later map these TDMs onto the molecular geometry of the MD simulations. Contrary to the PDI-C4, none of the Ru-bpyC9 TDMs aligns with a two-atom distance vector. We therefore introduce the two following vectors, derived as linear combinations of the nitrogen position vectors  (recall Figure 1):
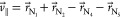
5
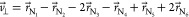
6where ∥ and ⊥ refers to parallel or perpendicular alignment *w.r.t.* the principal *C*_2_ axis of the complex, respectively. The resulting vectors are schematically presented in the inset in Figure 5b.

To assess the alignment of the TDMs with the  and  vectors defined above within the conformational ensemble, we compute the squared cosine of the included angle θ_*i*_ (see the inset in Figure 5b):
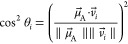
7

We use cos^2^ θ_*i*_, because cos^2^ θ_*i*_ + sin^2^ θ_*i*_ = 1. Since the cosine and sine of two angles different by 90° are the same, and, aside from distortions in the molecular geometry,  and  are approximately perpendicular to one another, we can use the identity cos^2^ θ_∥_ + cos^2^ θ_⊥_ ≈ 1.

The results of cos^2^ θ_∥_ and cos^2^ θ_⊥_ for the four transitions are displayed in Figure 5b for each of the 92 conformers of the ensemble. Now it is apparent that the TDMs in Ru-bpyC9 do not perfectly align with the vector based on atomic coordinates—different from the case for PDI-C4. The simplest case is the , as the TDMs of all the 92 conformers are quite close to , and the deviation is very small. For the excitation to S_5_, most of the conformers (81) have a TDM very well aligned with the , even if the deviation is more pronounced than in the S_6_ case. The transition dipole strengths of the  are quite small for some conformers, which means that the noise caused by geometrical distortions of the conformers has a greater influence relative to the dipole magnitude. This noise leads to a greater deviation in TDM orientations, such that the meaningfulness of the alignment is somewhat restricted. The disorder is even more significant for the transitions to the S_7_ and S_8_ states. However, when investigating one single conformer, more often than not one of the TDMs aligns with either  or , while the other TDM aligns with the respective other vector. It appears that these are indeed two states, one of which has a TDM aligned with , while the other aligns with , but due to geometrical distortions in the ensemble, the first state is not always lower in energy than the second. Rather, the two states are near-degenerate, and thus will be treated so as degenerated in the FRET computation. Thus, we assign them an averaged spectral overlap and transition intensity, even if they have opposite TDM orientations. To decide which TDM orientation belongs to which state, we use the most stable conformer (Figure 5a) and hence attribute  to  and  to . This is a reasonable decision as the most stable three conformers within the Ru-bpyC9 ensemble make up for 95% of the Boltzmann distribution, with the fourth adding another 4%. Compared to the overall ensemble, these three (or four) conformers exhibit TDMs that are reasonably well aligned with either of the reference vectors (Figure 5b). The worse aligned TDMs mostly occur for conformers with negligible weights, and we can conclude that  and  are well aligned with , and  and  orient along  as displayed in Figure 5a.

The TDM strength is scaled in the FRET rate computation in order to obtain the dipole strength expectation value of the ensemble Boltzmann weighted according to eq 4, resulting in  au^2^,  au^2^, and  au^2^, which correspond to 1.2, 2.5, and 3.2 D, respectively.

**Figure 6:**
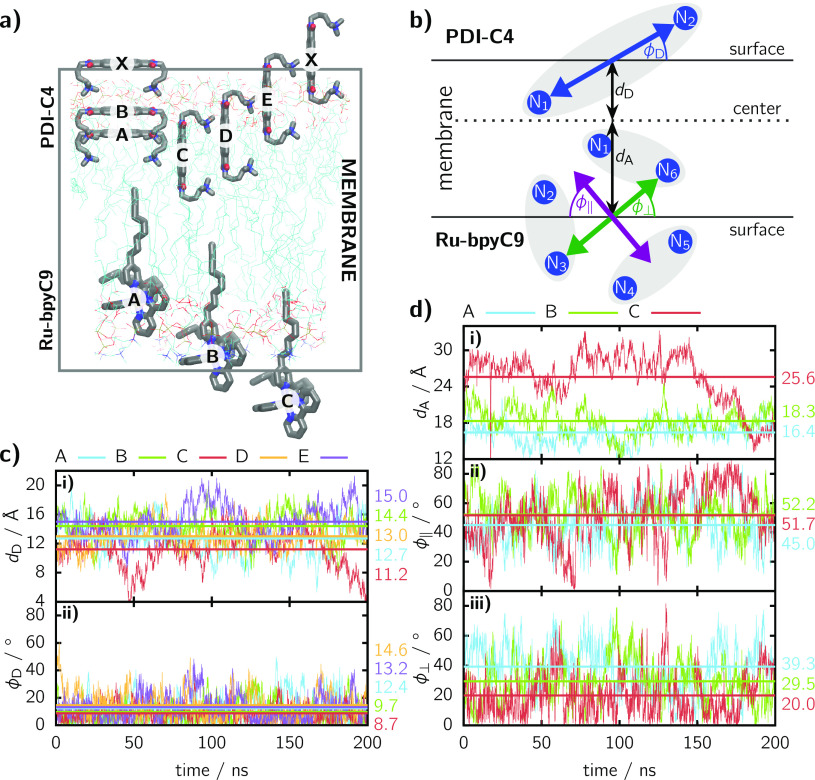
Molecular dynamics simulations of the chromophores in the membrane. (a) Insertion modes for the starting structures of PDI-C4 and Ru-bpyC9 in the membrane. (b) Definitions of the angles and distances of the corresponding TDMs within the membrane. (c) Time evolution of the distance *d*_D_ of PDI-C4 to the center of the membrane and of angle ϕ_D_. (d) Time evolution of the distance *d*_A_ of Ru-bypC9 to the center of the membrane and of angles ϕ_∥_ and ϕ_⊥_. In parts c and d, the trajectory mean values are indicated with horizontal lines and numbers on the right side of the plot.

The second step of this computational approach is to monitor the position and orientation of the chromophores and their TDMs within the actual membrane. To this aim, we perform classical MD simulations. We generate different reasonable starting positions of the chromophores within the membrane (see Figure 6a) and track the distance between the chromophore and the center of the membrane, *d*_*i*_, as well as ϕ_*i*_, the angle between the TDM and the surface of the membrane (see Figure 6b), throughout the MD simulations. Specifically, we generate seven starting positions for PDI-C4, so that three trajectories start with the aromatic plane of PDI-C4 aligned parallel to the surface of the membrane and four perpendicular to it (see Figure 6a). Each trajectory differs by its insertion depth, i.e., the distance to the membrane center, and we place PDI-C4 approximately in the hydrophobic part of the membrane, embedded into the polar head groups, or on the membrane–water interface. From both groups, the trajectories marked with “X”, with the largest distance to the membrane center, led to PDI-C4 detaching from the membrane and were therefore excluded from the analysis. The five remaining trajectories are labeled A through E.

Already during the heating and equilibration phases of the simulations, the trajectories where PDI-C4 was initially positioned perpendicular to the surface of the membrane (C–E) lead to a reorientation of the chromophore in a parallel fashion. Thus, in all five trajectories, ϕ_D_, i.e., the angle between the TDM and the surface of the membrane, is below 30° for most of the simulation time, resulting in an average angle of 11.7° (see Figure 6c(ii)). Additionally, all trajectories converge to similar insertion depths inside the membrane with an average distance *d*_D_ to the membrane center of 13.3 Å (see Figure 6c(i)).

For the acceptor Ru-bpyC9, we start with three different insertion depths, as we assume the aliphatic tails to be pointing toward the membrane center, which limits the orientational degrees of freedom. Again, we place the chromophore above, at the same depth and below the polar head groups. The Ru-bpyC9 simulations (see Figure 6d(i)) revealed two different positions of the chromophore within the membrane, one closer to the membrane center at a distance of around 17 Å (trajectories A and B), and a second one just below 30 Å. The latter is present dominantly for most of trajectory C, but after ca. 150 ns, the Ru-bpyC9 moves into the position present in trajectories A and B, indicating the possibility of the chromophore to visit both insertion depths. Based on our simulations, both insertion depths should be included in the analysis, which we do by averaging over all three trajectories; this results in a mean distance to the membrane center *d*_A_ of 20.1 Å.

These two possible insertion depths do not seem to significantly alter the angles of  and  relative to the surface of the membrane (see Figure 6d(ii,iii)), even if there is a lot of noise. In all three trajectories, neither  nor  are clearly oriented parallel or perpendicular to the surface of the membrane, but rather orient approximately as bisectors, with  averaging slightly above 45° at 49.6°, and  below that at 29.6°.

At this point, we have measured the magnitude (in the QM step) as well as the angle to the surface of the membrane (in the MD step) of all TDMs of the relevant excited states. Thus, we can now define representative vectors that have both the correct angle to the membrane surface as well as the fitting dipole strength to use these in the calculation of the interaction factor |*V*_DA_|^2^ (eq 1). We initially define all vectors in the *xz*-plane, i.e., the *y*-component is set to 0. This leads to the following vectors:
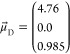

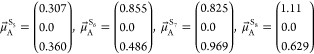


These definitions place all TDMs into the *xz*-plane. However, since the membrane merely confines the angle of the TDM toward its surface, it allows for free rotation within the x*y*-directions. For simplicity, we keep  fixed and apply a rotation operator **R** to the acceptor TDMs:
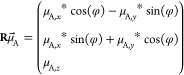
8

In our sampling, the rotation angle φ is step-by-step increased from 0° to 359° in steps of 1°. The FRET rate is computed at every point and averaged over all possible orientations.

Assuming that the chromophores are directly opposite in the membrane, the distance vector can be defined solely in the *z*-direction as the sum of the individual distances of the chromophore distances, such that *r*_*z*_ = 13.3 Å + 20.1 Å = 33.4 Å, recall Figure 6b. This assumption neglects any displacements of the chromophores along the surface of the membrane. Furthermore, relying on an averaged distance between the chromophores over the course of the simulation does not accurately represent how each individual possible distance influences the FRET rate. Since the rate scales inversely with the sixth power of the distance, an arithmetic mean as done here (where every distance is assigned the same weight) underestimates the influence of particular arrangements where the chromophores are close. However, this arithmetic mean does not imply an arbitrary error, but a systematic one; moreover, such a mean distance is easier to interpret than an averaged inverse sixth power distance. Therefore, here we deem the arithmetic mean as an adequate representation.

Using the TDMs defined above, together with the distance vector, allows for rotational sampling in order to compute the squared intermolecular electrostatic interactions |*V*_DA_|^2^ (eq 1). The results for the four TDM combinations are shown in Table 1.

**Table 1:** *k*_FRET_ Factors Computed from the *Two-Step* Approach

PDI-C4	Ru-bpyC9	|*V*_DA_|^2^ (kg^2^ m^4^ s^–4^)	|*V*_DA_| (cm^–1^)	*J* (cm)	*k*_FRET_ (s^–1^)	τ_FRET_ (ns)
S_1_ → S_0_	S_0_ → S_5_	9.90 × 10^–47^	0.501	2.12 × 10^–4^	6.28 × 10^7^	15.9
S_1_ → S_0_	S_0_ → S_6_	5.80 × 10^–46^	1.21	1.44 × 10^–4^	2.49 × 10^8^	4.01
S_1_ → S_0_	S_0_ → S_7_	7.15 × 10^–46^	1.35	8.00 × 10^–5^	1.72 × 10^8^	5.82
S_1_ → S_0_	S_0_ → S_8_	9.76 × 10^–46^	1.57	8.00 × 10^–5^	2.34 × 10^8^	4.27
overall					7.18 × 10^8^	1.39

The computed interaction values, |*V*_DA_|^2^, can be easily interpreted because (i) the donor–acceptor distance is identical for all combinations of donor and acceptor states, (ii) the pairs of S_0_ → S_5_ and S_0_ → S_7_ as well as S_0_ → S_6_ and S_0_ → S_8_ each have the same orientation of the TDM and differ only in TDM strength, and (iii) S_0_ → S_7_ and S_0_ → S_8_ are considered to have the same dipole strength due to their degeneracy and differ only in orientation. Accordingly, one can see that the interaction including the S_0_ → S_5_ transition is comparatively weak mostly because its low dipole strength, while the interaction involving the S_0_ → S_7_ is stronger due to its higher dipole strength, despite the same distance and orientation of the TDM. The same is true for the pair of S_0_ → S_6_ and S_0_ → S_8_, where the difference in |*V*_DA_|^2^ can be solely attributed to the reduced dipole strength of . Of particular interest is the difference in |*V*_DA_|^2^ between S_0_ → S_7_ and S_0_ → S_8_. The reduction of ^1^/_4_ when comparing the former to the latter is due to the less favorable alignment of TDMs.

From the computed Boltzmann weighted spectra (Figures 3 and 4), the spectral overlap between the (shifted) donor emission and acceptor absorption can be evaluated from eq 3. We note that the shifting of the emission spectrum naturally affects the magnitude of the spectral overlap. In this case, we are able to compare our TD-DFT results with experimental data, but in the absence of that it is also possible to benchmark the spectrum against a higher level of theory, so that the approach remains purely within the realm of theory.

The computed spectral overlaps *J* for the four pairs of transitions are presented in Table 1. Since the spectra were normalized to the unit area prior to the computation, the overlap diminishes for higher excitation energies in the acceptor. Therefore, the overlap is a relative indicator of the FRET rate, while any absolute values are represented in |*V*_DA_|^2^ in form of the intensities, i.e., dipole strengths. Plugging |*V*_DA_|^2^ and *J* into eq 2 yields the FRET rate *k*_FRET_ and the lifetime τ_FRET_ = 1/*k*_FRET_. Due to the comparatively small |*V*_DA_|^2^, the FRET rate for the S_0_ → S_5_ transition is one order of magnitude below the other three. These in turn are about equal in rate, with lifetimes in the nanosecond regime. Summation over all individual rates yields the overall FRET rate for the system, which corresponds to a lifetime of 1.39 ns. It has to be emphasized that the absolute values given are to be taken with a grain of salt, as we had to include several approximations within our model, for instance concerning the displacement of the chromophores along the membrane. Rather, these values allow for a relative evaluation of the FRET rate with respect to the different electronic excited states and orientations, as well as between the two approaches compared in this work.

### *One-Step* FRET Computation Directly in the Membrane

The influence of the membrane on the photophysical properties of the chromophores can be accurately assessed performing QM/MM excited state calculations. This approach additionally provides the orientation and magnitude of the TDMs directly in the membrane in one step.

**Figure 7:**
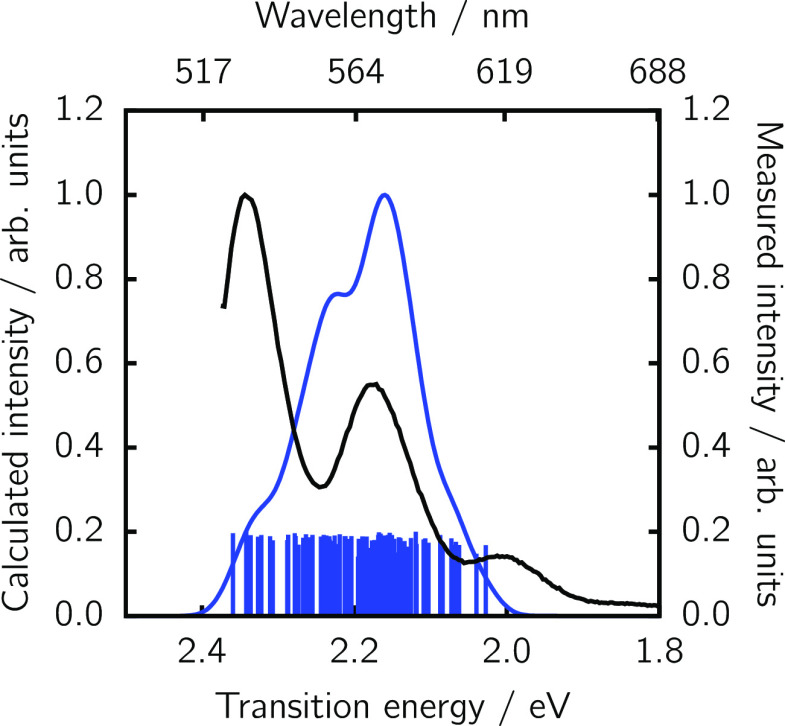
Computed QM/MM emission spectrum of the donor PDI-C4 (blue line), as obtained from 100 snapshots versus the experimental counterpart (black line). Individual S_1_ vertical excitation energies are shown with impulses.

The QM/MM emission spectrum of PDI-C4 within the membrane environment is presented in Figure 7, limited to the S_1_ → S_0_ transitions. These range from 2.0 to 2.4 eV for the different snapshots. Unfortunately, the convoluted spectrum does not resemble the fine structure of the experimental profile. This is because here we cannot use the FCHT approach to include vibrational modes, as we did in the *two-step* model before. Instead, we only rely on the MD generated ensemble that includes a classical vibrational sampling of the ground state mode. Unfortunately, the FCHT method is not easily compatible with dynamic QM/MM trajectories. Within the FCHT approach, the nuclear wave functions are approximated within the realm of a quantum harmonic oscillator and they are not limited to the vibrational ground state but include excitations of normal modes. So even if practically feasible, computing vibrationally resolved spectra with the FCHT method on snapshots taken from MD simulations would imply a repeated inclusion of the ground state sampling, which we do not consider physically sound. As the fine structure is not resolved, and therefore it is not apparent which excitations correspond to what band in the experimental counterpart, no shift was applied to the QM/MM emission spectrum of PDI-C4.

**Figure 8:**
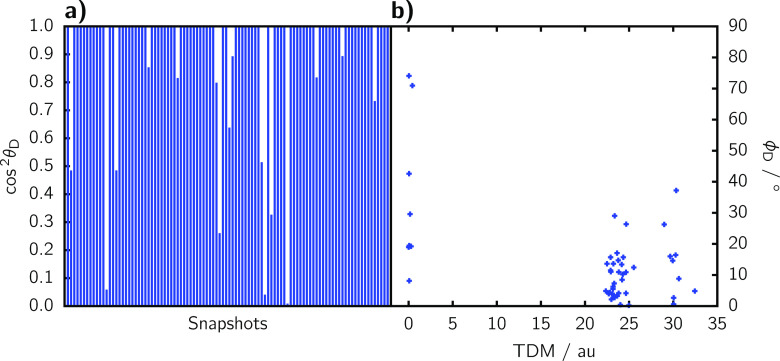
(a) cos^2^ θ_D_ for the S_1_ → S_0_ transition in the QM/MM snapshots of PDI-C4. (b) Angle of the TDM to the surface of the membrane *w.r.t.* the dipole strength.

We now compute the cos^2^θ_D_ to assess the alignment of  with the N–N distance vector  for the conformational ensemble. Most of the TDMs still align well with  (see Figure 8a); in fact, in 84 of the 100 snapshots, cos^2^ θ_D_ is larger than 0.992, which corresponds to an angle θ_D_ of less than 5°. In turn, also the angle to the surface of the membrane ϕ_D_ (recall Figure 6b) is comparable to that defined in the *two-step* approach. As visible from Figure 8b, with few exceptions, all TDMs of noteworthy intensity exhibit angles below 20°. Also the overall dipole strength is close to the Boltzmann weighted dipole strength expectation value from the implicit solvation calculations of 23.6 au^2^, with only some dipole strengths close to zero, probably caused by an excited state of different electronic character, that is more stabilized in some conformers than in the majority.

**Figure 9:**
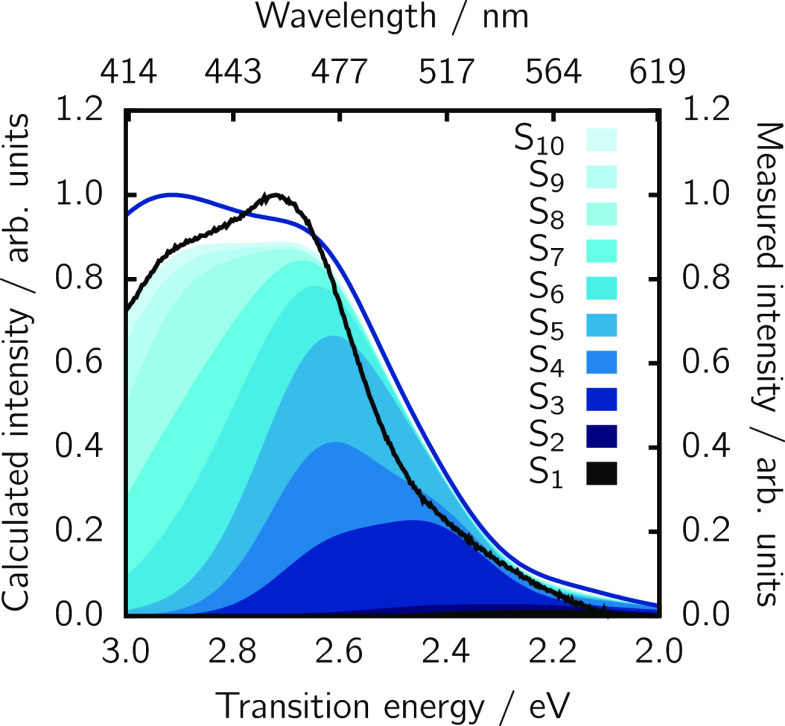
Computed QM/MM emission spectrum of the acceptor Ru-bpyC9 (blue line), as obtained from 100 snapshots compared to the experimental spectrum (in black). The contributions of the first 10 excited states are shown in shaded areas.

The overall QM/MM absorption spectrum of acceptor Ru-bpyC9 (see Figure 9) nicely resembles both the experimental as well as the implicit solvation spectrum. However, while in the latter case the spectrum is mostly composed of electronic transitions to the S_5_–S_8_ excited states, the QM/MM spectrum is more complicated as it results from transitions to all the lowest-lying ten electronic states. Above 2.6 eV, it becomes apparent that even higher lying states contribute to the spectrum, but since the donor emission spectrum levels off at 2.4 eV, we refrained from including higher lying excitations. For the FRET rate computation, we include all 10 electronic states.

**Figure 10:**
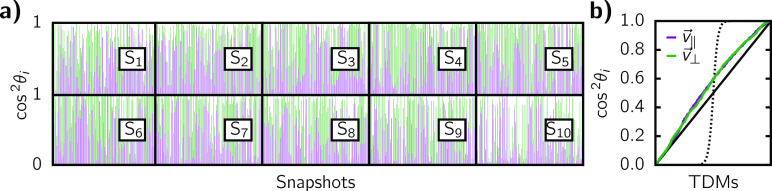
(a) cos^2^ θ_*i*_ for each of the transitions in the QM/MM calculations for Ru-bpyC9. Instead of by transitions S_0_ → S_*i*_, all TDMs are labeled by their final state. b) cos^2^θ_*i*_ for all ten lowest-lying excited singlet states sorted by alignment to either  or . For reference, a linear function (solid line) and a sigmoid curve (dotted line) are drawn.

Another difficulty of the QM/MM calculations of Ru-bpyC9 is to identify whether the TDMs are aligned along the  or  vectors. For none of the ten states, a dominant alignment with either vector can be identified (see Figure 10a). The geometrical distortions in the simulations even result in  or  not being perpendicular to one another, as apparent from cos^2^ θ_∥_ + cos^2^ θ_⊥_ being significantly different than 1 in many snapshots. The lack in alignment is easier to realize in Figure 10b. There, cos^2^ θ_∥_ and cos^2^ θ_⊥_ are condensed into one line each for all 10 states and the individual TDMs are sorted by their cos^2^ θ_*i*_ value, which allows one to quickly assess how well the TDMs are aligned to either  or . If the majority of TDMs exhibits dot products close to either 0 or 1, the expected curve would be of sigmoid shape, as indicated by the dotted line. However, it is evident that for both reference vectors the curves are mostly close to linear, indicating a random distributions of alignments.

**Figure 11:**
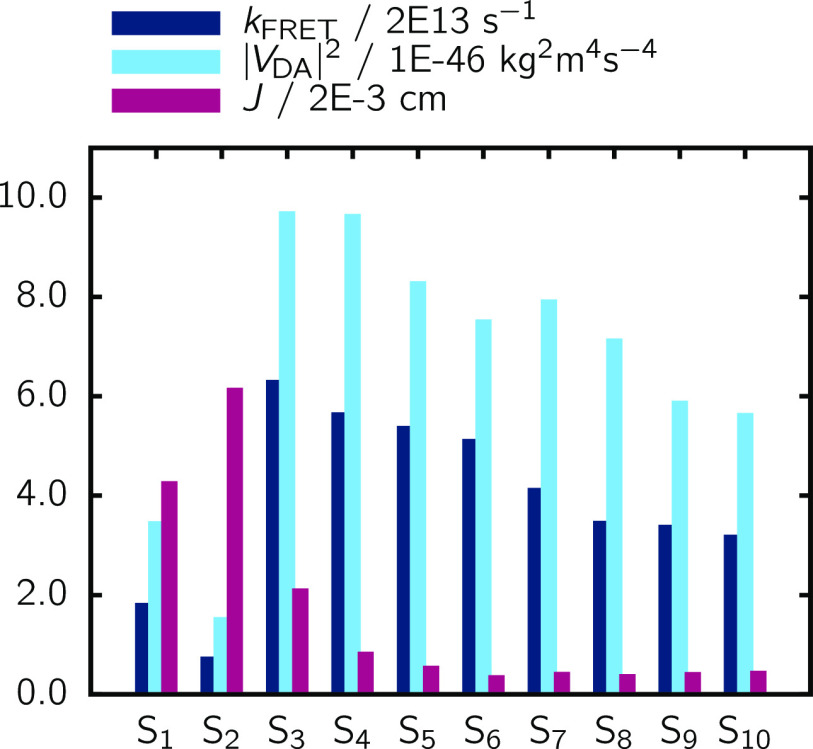
FRET rate *k*_FRET_, interaction factor |*V*_DA_|^2^ and spectral overlap *J* for each of the 10 states in Ru-bpyC9 averaged over all QM/MM snapshots.

As a consequence, the contributions to the FRET rate are of similar magnitude throughout all ten states (see Figure 11). Here, we compute |*V*_DA_|^2^ between all PDI-C4 snapshots and all Ru-bpyC9 snapshots individually, i.e., using directly the computed TDMs and the current distance at these snapshots. Again, we sample the rotational freedom according to eq 8 and compute the interchromophoric distance as the sum of the distances to the membrane center (recall Figure 6b) for each individual pair of snapshots. The spectral overlap is also computed on a per-pair basis as the overlap of two Gaussian functions each centered around the vertical excitation energy with a fwhm of 0.05 or 0.2 eV for PDI-C4 and Ru-bpyC9, respectively (the same fwhm used for the convolution of the spectra in Figures 7 and 9). By computing the factors influencing the FRET rate on a per-snapshot basis, the correct distances, TDMs and spectral overlaps are combined. In principle, it could be possible that certain orientations are only realized at specific distances or that shorter distances cause a specific shift in spectral overlap, as the environment closer to the center of the membrane is less polar. This per-snapshot computation correctly includes the effects of such scenarios on the energy transfer rate, which would have been lost when averaging the properties individually. Furthermore, in this *one-step* approach, there is no approximation included that could have been caused by the referencing of the TDMs onto the nuclear coordinates and by methodological inconsistencies arising from the averaging of distance and TDM orientation, as has been done in the *two-step* approach. Contrary to the *two-step* approach, we do not shift any of the spectra, as no better agreement between theory and experiment would result from that.

The spectral overlap *J* is largest for the S_0_ → S_2_ transition in Ru-bpyC9 (Figure 11) and quickly diminishes for the other states, as the excitation energy of S_0_ → S_1_ is too low for a better resonance with the PDI-C4 emission, while all other transitions occur at too high energies. However, the interaction factor |*V*_DA_|^2^ is lowest for S_0_ → S_1_ and S_0_ → S_2_, most likely due to the comparatively weak intensities of these bands (compare Figure 9), resulting in reduced FRET rates for these states. For the remaining eight states, both the interaction factor as well as the final rate diminish the higher the state is. It has to be pointed out, however, that the averaged *k*_FRET_ is not directly computed from the averaged interaction factors |*V*_DA_|^2^ and overlaps *J*, but rather from the individual rates each computed from the individual factors.

The interaction factors |*V*_DA_|^2^ presented in Figure 11 are of the same order of magnitude as those obtained from the *two-step* approach (compare Table 1). However, while in the *two-step* approach there were three states with a significant |*V*_DA_|^2^, here there are eight. Furthermore, what is not really represented in the averaged values is the fact that some states have high interaction factors of up to about 1 × 10^–44^ kg^2^ m^4^ s^–4^, mostly caused by very close interchromophoric distances. These go down to 21 Å, a distance significantly below the average value used in the *two-step* approach (33.4 Å). Still, these distances are well above any range where competing energy and charge transfers have to be taken into account, and this separation also justifies to investigate the excitations in PDI-C4 and Ru-bpyC9 individually. Furthermore, 21 Å represent the closest possible distance between the opposite chromophores, as in reality they could displace along the membrane–an effect that it is not considered here. The spectral overlaps, which range from 1.2 × 10^–2^ to 6.9 × 10^–4^ cm, appear also to be overestimated in comparison to the implicit solvation spectra. As a result, the true interaction factor will be smaller than what we report here.

Finally, we can compute an overall FRET rate by adding the individual rates of all 10 states and then averaging over all pairs of snapshots. This final FRET rate is 7.52 × 10^13^ s^–1^, which corresponds to a lifetime of 13.3 fs. This rate is dramatically higher than the one obtained from the *two-step* approach, which is mostly the result of the reduced distances and the overestimation of spectral overlaps in the *one-step* approach.

## Conclusions

In this work, we present two methods to compute FRET rates in lipid bilayers—a *two-step* approach, which combines photophysical data acquired from QM calculations with classical MM-MD simulations, and an integral approach that assesses the photophysical properties directly inside the environment employing QM/MM methodology in *one step*. The *two-step* approach offers detailed insight about the individual components contributing to the FRET, so that we are able to identify critical states in the acceptor and how their individual spectral overlaps and orientations affect the FRET efficiency.

The *one-step* QM/MM approach introduces dynamic sampling of geometries, which especially in the acceptor causes a broad distribution of all states both concerning excitation energy as well as the orientations of the TDMs. This broadening significantly hinders the identification of effects introduced by individual states. Rather, it becomes obvious that the necessary mapping of the TDMs done in the *two-step* approach only partly represents the true orientations and alignments in the Ru-bpyC9 acceptor. Because the TDM orientations in Ru-bpyC9 are comparably disordered, no effects of specific orientations on the FRET rate can be identified in the *one-step* approach. Instead, the final rate is rather an averaging over all possible alignments. By contrast, this mapping proved reasonably accurate for the more rigid PDI-C4 donor. On the one hand, this means that the approach of mapping TDMs onto the molecular geometry is of limited validity for flexible molecules. On the other hand, in order to exploit directional effects for the energy transfer rate optimization, more stiff and rigid systems than Ru-bpyC9 should be used.

The final FRET rates computed from the two approaches are 5 orders of magnitude apart. This means that none of these rates are accurate representations of the true energy transfer. The rate computed from the *one-step* approach can be regarded as an upper limit (fastest transfer), as we overestimate the contribution of the donor’s S_1_ state and we disregard the displacement of the chromophores along the membrane (thus assuming the closest distance between the chromophores). In the *two-step* approach, due to the distance and orientational averaging, we believe that the obtained rate constant is smaller (slower transfer) than the true one. Therefore, we expect the true rate to be in between, possibly in the range of picoseconds. Experimental measurements of perylene diimide-based chromophores, which closely resemble the here investigated PDI-C4 donor, recorded a fluorescence lifetime of around 5 ns.^42^ Both our approaches estimated shorter life times than fluorescence. Therefore, we anticipate that energy transfer for this system should be experimentally observable.

Both approaches, with their complementing strengths and drawbacks, provide valuable insight into what contributes to an efficient energy transfer. Here, we only employ our approach to one chromophore pair, but applying these approaches to different systems in a comparative fashion could enable the identification of more favorable ones *w.r.t.* the energy transfer efficiency. Thus, we hope that our methodology can be a basis for the identification of improved strategies toward the optimization of energy transfers in complex non-isotropic media.

## Supporting Information Available

Additional computational details, experimental details, and molecular orbitals (PDF)
Nuclear coordinates of the chromophores’ initial geometries (ZIP)
