# Supplementary material for: Computation of Förster Resonance Energy Transfer in Lipid Bilayer Membranes
Source: J Phys Chem A. 2022 Oct 19;126(43):8070–81. doi: 10.1021/acs.jpca.2c04524 (PMC9639162; doi:10.1021/acs.jpca.2c04524)
Supplement: Supplementary file 1 — jp2c04524_si_001.pdf [file jp2c04524_si_001.pdf]

Supporting Information to: On the Computation of Förster Resonance Energy Transfer in Lipid Bilayer Membranes

Richard Jacobi,[†,‡] David Hernández-Castillo,[†,‡] Novitasari Sinambela,[¶] Julian Bösking,[¶] Andrea Pannwitz,[¶] and Leticia González[*,†,§]

†*Institute of Theoretical Chemistry, Faculty of Chemistry, University of Vienna, Währinger Straße 17, 1090 Vienna, Austria*

‡*Doctoral School in Chemistry (DoSChem), University of Vienna, Währinger Straße 42, 1090 Vienna, Austria*

¶*Institute of Inorganic Chemistry I, Ulm University, Albert-Einstein-Allee 11, 89081 Ulm, Germany*

§*Vienna Research Platform on Accelerating Photoreaction Discovery, University of Vienna, Vienna, Austria, Währinger Straße 17, 1090 Vienna*

E-mail: leticia.gonzalez@univie.ac.at

Contents

S1 Additional computational details **S3**

S1.1 Generation of initial geometries S3

S1.2 Molecular dynamics simulations S4

S2 Experimental details **S7**

S2.1 Synthesis of chromophores S7

S2.1.1 Synthesis of N,N'-di(butylenedimethylamine)-3,4,9,10-perylenediimide S7

S2.1.2 Synthesis of N,N'-di(butylenetrimethylammonium)-3,4,9,10-perylenediimide S7

S2.2 Spectroscopy S8

S3 Molecular orbitals **S9**

S3.1 PDI-C4 . . . . S9

S3.2 Ru-bpyC9 . . . . S9

References **S11**

S1 Additional computational details

S1.1 Generation of initial geometries

Long aliphatic chains can adopt numerous different conformations, rendering it insufficient to merely compute the properties of one conformer.[1–3] Therefore, structures representative of the thermally-accessible conformational ensemble for PDI-C4 and Ru-bpyC9 are generated using the conformer-rotamer ensemble sampling tool (CREST).[4,5] In CREST, the generated structures are optimized using the geometry, frequency, non-covalent extended tight binding model of version 2 (GFN2-xTB).[6] For the sake of computational efficiency, the structure crossing step within the standard CREST workflow, which generates new conformers as difference structures between two already existing conformers,[7] is omitted for the Ru-bpyC9 calculation. Where noted, solvent effects are included implicitly using the analytical linearized Poisson-Boltzmann model.[4,5]

Usually, it is sufficient to consider all conformers within an energy range of 3 kcal/mol with respect to the global minimum as —according to the Boltzmann distribution for an idealized, non-degenerate two level system — the population of a conformer that is 3 kcal/mol less stable compared to the lowest energy conformer, would be well below 1%. However, to compensate for inaccuracies in the semiempirical tight-binding GFN2-xTB method on which the conformers are optimized and energies are computed within the CREST workflow, all conformers within an energy window of 7 kcal/mol are included in the initial steps of the ensemble generation.

To reduce the vast ensembles generated with CREST (>38,000 conformers for Ru-bpyC9, >2,000 structures for PDI-C4) without losing relevant structures, the principal component analysis and k-means clustering algorithm included in the CREST program package are employed, where the ensemble is grouped based on the geometry of the molecules. By only preserving one representative structure per cluster, the conformer number within the ensemble is decreased to 1000. Single point energies on these 1000 structures are then refined

at the BP86[8,9]/def2-SVP[10,11] level of theory using the quantum chemistry program ORCA (version 4.2.1).[12] Solvent effects are included implicitly using the polarizable conductor-like calculation model. The resolution of identity approximation with the auxiliary basis set def2/J is used. Based on the BP86/def2-SVP energies, the number of representative structures is reduced to a final 100 for PDI-C4, and 92 for Ru-bpyC9, respectively. Prior to computing the excited states for these representative structures, the geometries are re-optimized utilizing the more accurate B3LYP[9,13]/def2-SVP setup described below.

S1.2 Molecular dynamics simulations

All MD simulations are performed using the program packages Amber20 and AmberTools21.[14] Starting structures are selected for the chromophores (PDI-C4 and Ru-bpyC9) from the ensemble optimized on the B3LYP/def2-SVP level of theory. Point charges for the chromophores are computed using the restrained electrostatic potential atomic partial charges (RESP) scheme in Gaussian 16.[15] All point charges are computed on the optimized ground state geometries, such that, especially for the energy donor, any structural relaxations are neglected. The chromophores are described using the Generalized Amber Force Field (GAFF) included in AmberTools21, with additional parameters for the ruthenium complex developed by Brandt and coworkers[16] and used later by others.[17,18] The lipids are described using the Lipid17 force field, while water and the atomic ions are described using the ”optimal” point charge model[19] implemented in AmberTools21.

The lipid bilayer is assembled using the membrane builder of the web-based input generator CHARMM-GUI.[20] The membrane comprises one leaflet of DPPC lipids, the other leaflet of DOPG lipids, and is assembled in the xy-plane. The number of lipids is chosen automatically according to the box dimensions of 80 Å side length in the xy-plane. The resulting numbers vary depending on the chromophore and the insertion mode from 99 to 102 for DPPC and from 93 to 95 for DOPG.

To define the box dimension in z-direction, the hydration number of each lipid is set to

50, which is well in excess of experimental hydration numbers for similar lipids of 32.8.[21] The negative charge of the lipid head groups is neutralized by placing potassium ions into the solvated area of the box using the Monte-Carlo placing method, and subsequently the number of potassium and chlorine ions is increased to yield a salt concentration of 0.15 mol/L.

PDI-C4 is placed in the DOPG leaflet, Ru-bpyC9 in the DPPC leaflet. We generate multiple trajectories to account for different starting positions and orientations of each individual chromophore within their respective leaflet and propagate each for 200 ns while monitoring the chromophore distance to the membrane center as well as the angle of the TDMs *w.r.t.* the surface of the membrane.

Each system obtained from CHARMM-GUI is minimized using the Amber module pmemd in 5,000 minimization cycles employing a steepest descent algorithm, and another 5,000 additional steps using a conjugate gradient algorithm. The minimization is performed at constant pressure using anisotropic pressure scaling.

For all the MD simulations, a time step of 2 fs is used. To achieve this large time step, the SHAKE algorithm is turned on to freeze hydrogen bond lengths at a relative geometrical tolerance of 1e-7. Constant pressure periodic boundary conditions are superimposed with anisotropic pressure scaling. The cutoff for non-bonded interaction terms is set to 10 Å. The simulation is performed using the GPU (CUDA) version of pmemd.[22–24]

The minimized system is subsequently heated using the Langevin thermostat at a collision frequency of 1.0 ps^{-1} to 100 K in 2,500 time steps (5 ps), followed by a heating to 300 K in 50,000 steps (100 ps). The first heating is performed at a pressure relaxation time of 1 ps, which is increased to 2 ps for the second heating phase only. Prior to production, the system is equilibrated by running 10 ps of simulation time (5,000,000 time steps).

The analysis of the simulation trajectories is performed using the program CPPTRAJ.[25] Visualization is done using the molecular viewer VMD.[26] The distance to the center of the membrane is computed as the center-of-mass distance between the core atoms of the chromophores, *i.e.*, all non-hydrogen atoms excluding the aliphatic tails, and the C_{18} atoms of

the DOPG lipids. The C_{18} atoms are suitable for defining the center of the membrane, as they are the final carbon atoms in the hydrophobic tail, and thus can be expected to be furthest away from the membrane surface. To ensure that this corresponds to the minimum distance between the chromophore and the membrane center and not some artificially elongated distance misrepresented by lateral movement of the ligand within the membrane, the chromophore is imaged into the center of the periodic box for each frame during the analysis. In all systems, the membrane is assembled and remained in the xy-plane, so that the angle to the surface of the membrane is evaluated as the angle *w.r.t.* the xy-plane.

S2 Experimental details

S2.1 Synthesis of chromophores

The Ru-bpyC9 complex was synthesized according to a previously published procedure.[27] The synthesis of PDI-C4 was adapted from a similar compound and is reported below.[28]

S2.1.1 Synthesis of N,N'-di(butylenedimethylamine)-3,4,9,10-perylenediimide

In 20 mL of isobutanol PTCDA (0.1 g, 0.25 mmol, 1 eq.) and (4-aminobutyl) dimethylamines (0.14 mL, 1.02 mmol, 4 eq.) were combined, stirred and heated at 90°C for 24 h under argon atmosphere. After the mixture was cooled to room temperature, the crude product was collected by filtration. To remove unreacted PTCDA, to the mixture was added 5% aqueous NaOH solution and stirred at 90°C for 30 minutes. The product was separated from solvent by filtration and washed with water and ethanol. The red solid powder was then dried under vacuum to give 0.14 g (95% yield) product.

^{1}H NMR (400 MHz, CDCl3) δ 8.61 (d, J = 8.0 Hz, 4H), 8.53 (d, J = 8.1 Hz, 4H), 4.20 (t, 4H), 2.45 — 2.23 (m, 4H), 2.22 (s, 12H), 1.86 – 1.67 (m, 4H), 1.61 – 1.39 (m, 4H).

MALDI-MS: calcd. for $C_{36}H_{36}N_4O_4$ (m/z): 588.27, found: 588.27

Anal. Calcd.: C, 73.45; H, 6.16; N, 9.52; O, 10.87, found C, 73.16; H, 6.15; N, 9.49; O, 10.96.

S2.1.2 Synthesis of N,N'-di(butylenetrimethylammonium)-3,4,9,10-perylenediimide

A degassed solution of N,N'-di(butylenedimethylamine)-3,4,9,10-perylenediimide (0.1 g, 0.17 mmol, 1 eq.) and methyl iodide (0.3 mL, 1.70 mmol, 10 eq.) in 10 mL of toluene was refluxed for 3 h under argon atmosphere. The suspended mixture was collected by filtration, washed with ether and dried under vacuum. To the aqueous solution of obtained compound was added dropwise aqueous solution of NH_4PF_6. After stirring of the solution at 65°C for 1 h, the precipitate was centrifugated, washed with water to remove produced exchanges salts

and dried under vacuum to give 0.12 g (96% yield) product.

^{1}H NMR (400 MHz, CD_3CN) δ 8.47 – 8.30 (m, 8H), 4.18 (t, J = 6.7 Hz, 4H), 3.39 – 3.29 (m, 4H), 3.04 (s, 18H), 1.85 – 1.70 (m, 8H).

MALDI-MS: calcd. for $C_{3}8H_{4}2F_{6}N_{4}O_{4}P^{2+}$ (m/z): 763.28, found: 763.28

Anal. Calcd.: C, 59.76; H, 5.54; F, 14.93; N, 7.34; O, 8.38; P, 4.06, found C, 49.53; H, 4.81; F, 24.71; N, 6.07; O, 7.96; P, 6.74.

S2.2 Spectroscopy

Absorption spectra were recorded on a V-670 absorption spectrometer from JASCO. Starna fluorescence quartz cuvettes with a path length of 1 cm were used. Emission of PDI-C4 was recorded on a Horiba Jobin Y von FluoroMax4 Plus C spectrophotometer equipped with a 150 W xenon lamp, excitation range of 250 – 600 nm, emission range 500 – 700 nm. Spectra of PDI-C4 were recorded in acetonitrile, Ru-bpyC9 in the presence of DPPC liposomes in aqueous solution.

S3 Molecular orbitals

S3.1 PDI-C4

Both in the case of absorption (ground state geometry) and emission (S_1 excited state geometry), the $S_0 \rightarrow S_1$ transition is composed of 50% HOMO→LUMO character, while all other contributions are below 1%. The HOMO and LUMO orbitals are displayed below in top view and side view. Molecular orbitals are shown at a cut-off value of 0.03 a.u.

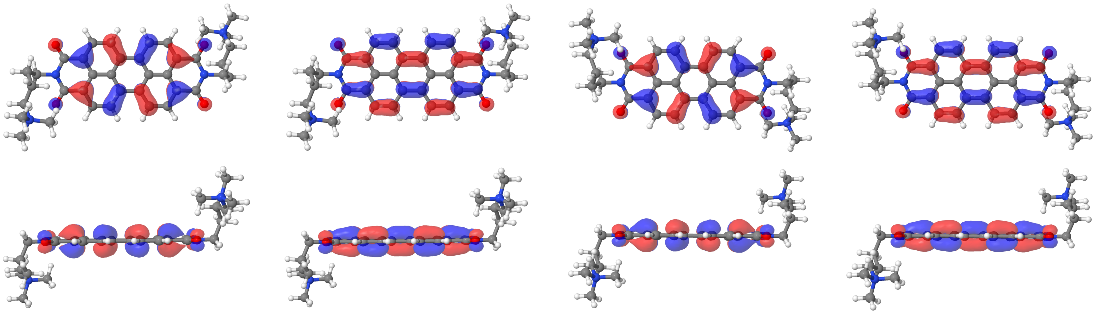

**HOMO**	**LUMO**	**HOMO**	**LUMO**
ground state geometry	ground state geometry	S_1 excited state geometry	S_1 excited state geometry

Figure S1: HOMO and LUMO of PDI-C4 on ground state and S_1 excited state geometry.

As visible from the orbitals, the $S_0 \rightarrow S_1$ transition is of $\pi\pi^*$ character.

S3.2 Ru-bpyC9

The transitions from S_0 to S_5, S_6, S_7, and S_8 are mainly composed of combinations of the highest three occupied molecular orbitals, to the lowest three unoccupied MOs. These are shown below. In the HOMOs, electron density is mostly localized on the metal center, while the LUMOs are delocaized over the ligands. As a result, all transitions are of metal to ligand charge transfer character.

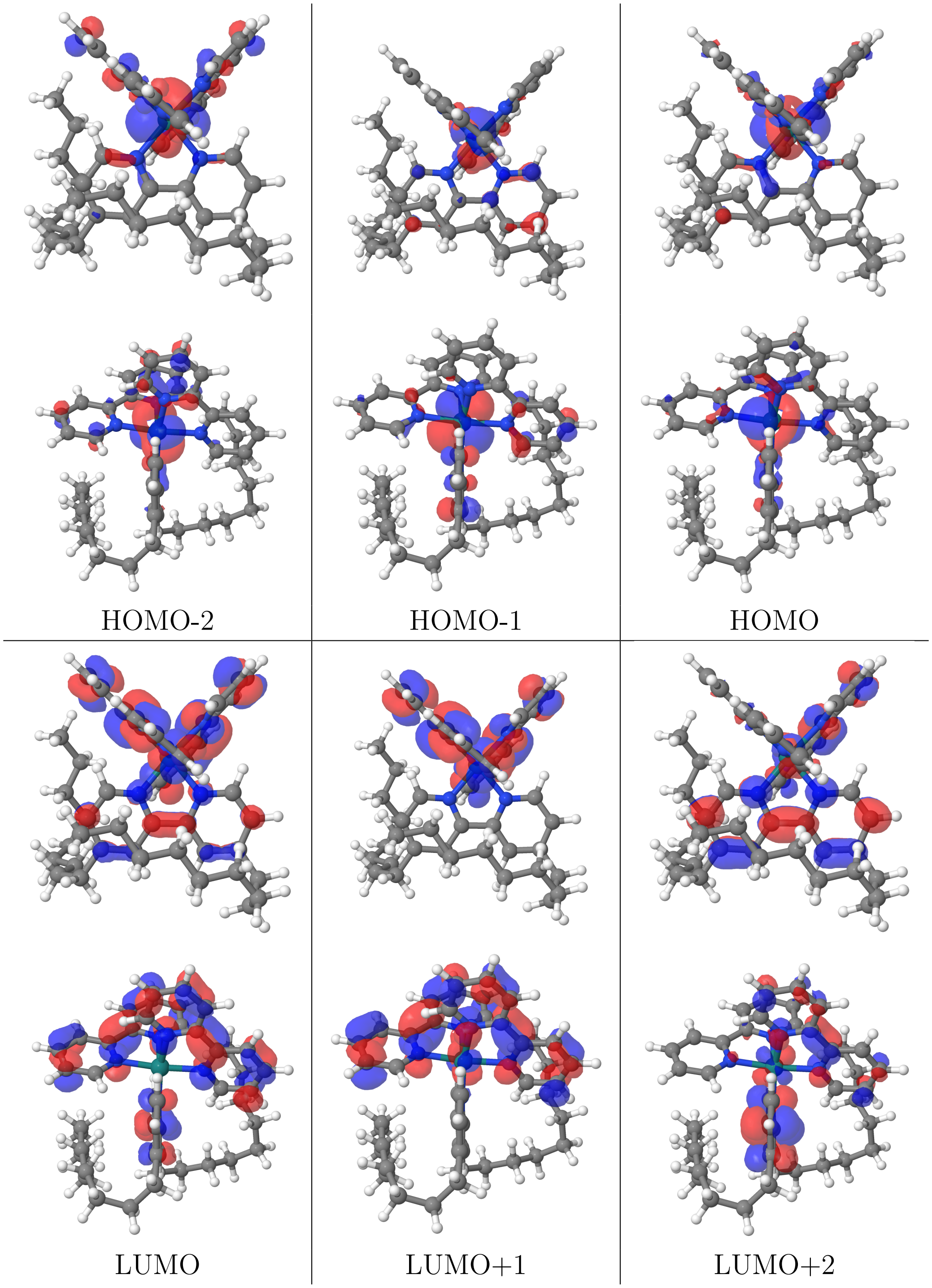

Figure S2: Highest three occupied and lowest three unoccupied MOs of Ru-bpyC9.

References

(1) Leach, A. R. *Molecular modelling: principles and applications*, 1st ed.; Longman: Harlow, 1996.

(2) Ebejer, J.-P.; Morris, G. M.; Deane, C. M. Freely Available Conformer Generation Methods: How Good Are They? *J. Chem. Inf. Model.* **2012**, *52*, 1146–1158.

(3) Hawkins, P. C. D. Conformation Generation: The State of the Art. *J. Chem. Inf. Model.* **2017**, *57*, 1747–1756.

(4) Grimme, S. Exploration of Chemical Compound, Conformer, and Reaction Space with Meta-Dynamics Simulations Based on Tight-Binding Quantum Chemical Calculations. *J. Chem. Theory Comput.* **2019**, *15*, 2847–2862.

(5) Pracht, P.; Bohle, F.; Grimme, S. Automated exploration of the low-energy chemical space with fast quantum chemical methods. *Phys. Chem. Chem. Phys.* **2020**, *22*, 7169–7192.

(6) Bannwarth, C.; Ehlert, S.; Grimme, S. GFN2-xTB—An Accurate and Broadly Parametrized Self-Consistent Tight-Binding Quantum Chemical Method with Multipole Electrostatics and Density-Dependent Dispersion Contributions. *J. Chem. Theory Comput.* **2019**, *15*, 1652–1671.

(7) Grimme, S.; Bannwarth, C.; Dohm, S.; Hansen, A.; Pisarek, J.; Pracht, P.; Seibert, J.; Neese, F. Fully Automated Quantum-Chemistry-Based Computation of Spin-Spin-Coupled Nuclear Magnetic Resonance Spectra. *Angew. Chem. Int. Ed.* **2017**, *56*, 14763–14769.

(8) Becke, A. D. Density-functional exchange-energy approximation with correct asymptotic behavior. *Phys. Rev. A* **1988**, *38*, 3098–3100.

(9) Perdew, J. P. Density-functional approximation for the correlation energy of the inhomogeneous electron gas. *Phys. Rev. B* **1986**, *33*, 8822–8824.

(10) Weigend, F.; Ahlrichs, R. Balanced basis sets of split valence, triple zeta valence and quadruple zeta valence quality for H to Rn: Design and assessment of accuracy. *Phys. Chem. Chem. Phys.* **2005**, *7*, 3297.

(11) Weigend, F. Accurate Coulomb-fitting basis sets for H to Rn. *Phys. Chem. Chem. Phys.* **2006**, *8*, 1057.

(12) Neese, F.; Wennmohs, F.; Becker, U.; Riplinger, C. The ORCA quantum chemistry program package. *J. Chem. Phys.* **2020**, *152*, 224108.

(13) Lee, C.; Yang, W.; Parr, R. G. Development of the Colle-Salvetti correlation-energy formula into a functional of the electron density. *Phys. Rev. B* **1988**, *37*, 785–789.

(14) Case, D.; Aktulga, H.; Belfon, K.; Ben-Shalom, I.; Brozell, S.; Cerutti, D.; Cheatham, T.; Ill,; Cruzeiro, V.; Darden, T. et al. Amber 2021. 2021.

(15) Frisch, M. J.; Trucks, G. W.; Schlegel, H. B.; Scuseria, G. E.; Robb, M. A.; Cheeseman, J. R.; Scalmani, G.; Barone, V.; Petersson, G. A.; Nakatsuji, H. et al. Gaussian16 Revision C.01. 2016.

(16) Brandt, P.; Norrby, T.; Åkermark, B.; Norrby, P.-O. Molecular Mechanics (MM3*) Parameters for Ruthenium(II)-Polypyridyl Complexes. *Inorg. Chem.* **1998**, *37*, 4120–4127.

(17) Moret, M.-E.; Tavernelli, I.; Rothlisberger, U. Combined QM/MM and classical molecular dynamics study of $[Ru(bpy)_3]^{2+}$ in water. *J. Phys. Chem. B* **2009**, *113*, 7737–7744.

(18) Sánchez-Murcia, P. A.; Nogueira, J. J.; González, L. Exciton Localization on Ru-Based Photosensitizers Induced by Binding to Lipid Membranes. *J. Phys. Chem. Lett.* **2018**, *9*, 683–688.

(19) Izadi, S.; Anandakrishnan, R.; Onufriev, A. V. Building Water Models: A Different Approach. *J. Phys. Chem. Lett.* **2014**, *5*, 3863–3871.

(20) Jo, S.; Kim, T.; Iyer, V. G.; Im, W. CHARMM-GUI: A web-based graphical user interface for CHARMM. *J. Comput. Chem.* **2008**, *29*, 1859–1865.

(21) Kučerka, N.; Nieh, M.-P.; Katsaras, J. Fluid phase lipid areas and bilayer thicknesses of commonly used phosphatidylcholines as a function of temperature. *Bba-biomembranes* **2011**, *1808*, 2761–2771.

(22) Salomon-Ferrer, R.; Götz, A. W.; Poole, D.; Grand, S. L.; Walker, R. C. Routine Microsecond Molecular Dynamics Simulations with AMBER on GPUs. 2. Explicit Solvent Particle Mesh Ewald. *J. Chem. Theory Comput.* **2013**, *9*, 3878–3888.

(23) Götz, A. W.; Williamson, M. J.; Xu, D.; Poole, D.; Grand, S. L.; Walker, R. C. Routine Microsecond Molecular Dynamics Simulations with AMBER on GPUs. 1. Generalized Born. *J. Chem. Theory Comput.* **2012**, *8*, 1542–1555.

(24) Grand, S. L.; Götz, A. W.; Walker, R. C. SPFP: Speed without compromise—A mixed precision model for GPU accelerated molecular dynamics simulations. *Comput. Phys. Commun.* **2013**, *184*, 374–380.

(25) Roe, D. R.; Cheatham, T. E. PTRAJ and CPPTRAJ: Software for Processing and Analysis of Molecular Dynamics Trajectory Data. *J. Chem. Theory Comput.* **2013**, *9*, 3084–3095.

(26) Humphrey, W.; Dalke, A.; Schulten, K. VMD: Visual molecular dynamics. *J. Mol. Graphics* **1996**, *14*, 33–38.

(27) Klein, D. M.; Rodríguez-Jiménez, S.; Hoefnagel, M. E.; Pannwitz, A.; Prabhakaran, A.; Siegler, M. A.; Keyes, T. E.; Reisner, E.; Brouwer, A. M.; Bonnet, S. Shorter Alkyl

Chains Enhance Molecular Diffusion and Electron Transfer Kinetics Between Photosensitisers and Catalysts in CO2-Reducing Photocatalytic Liposomes. *Chem. Eur. J.* **2021**,

(28) Berardi, S.; Cristino, V.; Canton, M.; Boaretto, R.; Argazzi, R.; Benazzi, E.; Ganzer, L.; Varillas, R. B.; Cerullo, G.; Syrgiannis, Z. et al. Perylene Diimide Aggregates on Sb-Doped SnO_2: Charge Transfer Dynamics Relevant to Solar Fuel Generation. *J. Phys. Chem. C* **2017**, *121*, 17737–17745.
